# Cytosine Methylation Within Marine Sediment Microbial Communities: Potential Epigenetic Adaptation to the Environment

**DOI:** 10.3389/fmicb.2019.01291

**Published:** 2019-06-11

**Authors:** Ian M. Rambo, Adam Marsh, Jennifer F. Biddle

**Affiliations:** School of Marine Science and Policy, University of Delaware, Lewes, DE, United States

**Keywords:** epigenetics, methylation, metagenome, sediment, chitinase, transposase

## Abstract

Marine sediments harbor a vast amount of Earth’s microbial biomass, yet little is understood regarding how cells subsist in this low-energy, presumably slow-growth environment. Cells in marine sediments may require additional methods for genetic regulation, such as epigenetic modification *via* DNA methylation. We investigated this potential phenomenon within a shallow estuary sediment core spanning 100 years of age. Here, we provide evidence of dynamic community m5-cytosine methylation within estuarine sediment metagenomes. The methylation states of individual CpG sites were reconstructed and quantified across three depths within the sediment core. A total of 6,254 CpG sites were aligned for direct comparison of methylation states between samples, and 4,235 of these sites mapped to taxa and genes. Our results demonstrate the presence of differential methylation within environmental CpG sites across an age gradient of sediment. We show that epigenetic modification can be detected *via* Illumina sequencing within complex environmental communities. The change in methylation state of environmentally relevant genes across depths may indicate a dynamic role of DNA methylation in regulation of biogeochemical processes.

## Introduction

Marine sediments are some of the largest reservoirs of microbial biomass on Earth (Whitman et al., 1998; Kallmeyer et al., 2012), and describing the relationships between community structure, activity, and ecosystem function in these habitats remains a challenge (Biddle et al., 2008; Fuhrman, 2009; Orsi et al., 2013; Zinke et al., 2017). The physiological states representative of sedimentary bacteria and archaea in their natural habitats are unknown (Hoehler and Jørgensen, 2013). Determining the drivers that govern microbial activity in the subsurface is a key to understanding the relationships between these communities and their environments. Models of the marine subsurface suggest that biomass turnover rates are on the scale of thousands of years and assume that many marine subsurface microbes have formed spores due to the low availability of energy (Lomstein et al., 2012) and endospores have been found to be globally significant in the deep biosphere (Wormer et al., 2019), yet metagenomic analyses of deep-sea sediment communities exhibit low observed frequencies of endospore-specific genes (Kawai et al., 2015). Isolates obtained from the deep biosphere are genetically similar to members of surface communities (Inagaki et al., 2015; Russell et al., 2016), suggesting that microbial cells able to adapt to the subsurface possibly suspend certain life processes to subsist at low levels of activity without global genetic changes. Epigenetic mechanisms may offer potential microbial survival strategies within low-energy sediment, allowing for cell maintenance and acclimation to environmental stressors (Bird, 2002; Casadesús and Low, 2006; Low and Casadesús, 2008).

DNA methylation is a conserved epigenetic modifier in prokaryotes whose roles include gene regulation and defense against invading foreign DNA or restriction-modification (RM) (Low et al., 2001; Wion and Casadesús, 2006; Brunet et al., 2011; Kumar and Rao, 2012; Beaulaurier et al., 2018). It involves the addition of a methyl group *via* a DNA methyltransferase (MTase) to either the carbon 5 position of a cytosine [resulting in 5-methylcytosine (m5C)], the nitrogen 4 position of a cytosine [resulting in N4-methylcytosine (m4C)], or the nitrogen 6 position of an adenine [resulting in N6-methyladenine (m6A)] within a specific nucleotide target sequence (Ratel et al., 2006). These modified bases comprise an organism’s methylome and are generally formed by two different MTase activities: “maintenance” and “*de novo*” methylation (Bird, 2002; Kuhlmann et al., 2005). While DNA methylation is an integral part of RM systems involved in the recognition of self vs. non-self DNA for cellular defense and mismatch repair during DNA replication, growing evidence indicates that prokaryotes can utilize both adenine and cytosine methylation as a means of regulating gene expression (Low et al., 2001; Reisenauer and Shapiro, 2002; Løbner-Olesen et al., 2005; Srikhanta et al., 2005, 2009; Wion and Casadesús, 2006; Low and Casadesús, 2008; Collier, 2009; Marinus and Casadesús, 2009; Brunet et al., 2011; Kahramanoglou et al., 2012; Gonzalez et al., 2014; Blow et al., 2016; Walworth et al., 2017; Hiraoka et al., 2019). Individual genes have been shown to be under methylation control, for example, the *pap* operon in *Escherichia coli*, and cellular cycle events have also been shown to be controlled by methylation events in the Gammaproteobacteria (Casadesús and Low, 2006). RNA polymerase, transcription factors, and binding proteins are able to recognize the methylated states of modified bases within target genes, and this discrimination of differentially methylated DNA acts as a method for determining which genes are transcribed (Low and Casadesús, 2008; Collier, 2009; Gonzalez et al., 2014). Recently, methylation state was tied to transcriptional activity that had an imprint of biogeography on cultivated strains, showing that microbial cells may employ long-term methylation signatures as regulatory elements for ecophysiological adaptation (Walworth et al., 2017).

Nucleotide base modifications within the methylome exist in a more dynamic system compared to the nucleotide composition of an organism’s genome, exhibiting plasticity outside of binary “methylated” or “non-methylated” states (Ichida et al., 2007; Chernov et al., 2015). A system of genetic “switches” (Hernday et al., 2004) regulated by dynamic DNA methylation could be a viable mechanism for both long-term and short-term transcriptional silencing for microbes inhabiting marine sediments, allowing adaptation to slow growth without additional genetic content. Initial examinations into environmental epimetagenomes have used single-molecule real-time sequencing to detect methylated bases (Walworth et al., 2017; Hiraoka et al., 2019); however, this method is limited by input DNA and the need for extremely deep read depth for methylation detection. We utilized a methylation-sensitive restriction enzyme-based Illumina sequencing assay to identify dynamic shifts in CpG methylation within sediment metagenomes across varying ages from the Broadkill River. We saw that methylation signatures shifted from mostly methylated to a bimodal state of methylation with increasing sediment depth and that environmentally relevant genes showed distinct methylation signatures, suggesting that cytosine methylation may be used as a genetic regulation signature in marine sediments. This is the first report on DNA methylation within sediment metagenome sequence data and is the first to utilize this Illumina-based CpG methylation assay in an environmental application.

## Materials and Methods

### Core Collection

Sediment cores were sampled from the Oyster Rocks site of the Broadkill River, Milton, DE, USA (38.802161, −75.20299) at low tide in July 2012 and 2014. The 2012 core was sectioned into 3 cm sections and immediately frozen at −80°C. The sediment collection from 2012 was depleted to extract sufficient DNA for sequencing. Three cores were extracted from the same site in 2014: a 32 cm radionuclide dating core (R), and 25 cm (S), and 30 cm (L) cores for pore water ion chromatography, methane flame ionization gas chromatography, and porosity measurements. Cores L and S were sliced into 3 cm depth samples and frozen at −80°C. Core R was immediately processed.

### Radionuclide Dating

Core R was sectioned into 1-cm thick intervals from 0 to 10 cm and 2-cm thick intervals from 10 to 32 cm. Samples were weighed, dried at 60°C for 48 h, reweighed, and transferred to a 25°C desiccation chamber. Dried samples were crushed and then ground into a fine powder with an IKA Werke M20 mill (IKA Werke, Staufen, Germany). Radionuclide counting of compressed samples was performed for 24 h on a Canberra Instruments Low Energy Germanium Detector (Canberra Industries, Meriden, CT, USA). Levels of ^7^Be (*t*_1/2_ = 53.22 days), ^210^Pb (*t*_1/2_ = 22.20 years), and ^137^Cs (*t*_1/2_ = 30.17 years) activity were measured by gamma spectroscopy of the 478, 46.5, and 662 keV photopeaks, respectively (Cutshall et al., 1983; Igarashi et al., 1998; Wallbrink et al., 2002).

### Geochemistry

Porewater was extracted from 50 ml sediment samples by centrifugation. Porewater ions were measured with a Metrohm 850 Professional ion chromatograph (Metrohm, Herisau, Switzerland). Methane concentrations were determined for Core L and S subsamples as previously described (Biddle et al., 2012). Mean headspace methane concentrations were determined in triplicate *via* flame ionization gas chromatography using a 5890 Series II gas chromatograph equipped with a flame ionization detector (Hewlett-Packard, Palo Alto, California, USA).

### Metagenome Library Preparation and Sequencing

Metagenome libraries were prepared from three of the 2012 sediment core sections (3–6, 12–15, and 24–27 cm). Genomic DNA (gDNA) was extracted from 0.5 g of wet sediment from each core section with a MoBio PowerSoil (MoBio, Valencia, CA) kit per the manufacturer’s protocol, and 15 individual extractions were pooled. A 10 μg aliquot of purified gDNA was digested with the methylation-sensitive restriction endonuclease HpaII, which cleaves at the unmodified internal cytosine of a 5′-CCGG-3′ motif. The restriction enzyme approach introduces a methylation-dependent fragment distribution into the gDNA library, significantly enriching the metagenome for their presence. Digested DNA was cleaned with a QIAquick PCR purification kit (Qiagen, Hilden, Germany), sheared to a median size of 300 bp using a Covaris focused-ultrasonicator (Covaris, Woburn, MA, USA), and cleaned again with QIAquick. Illumina libraries were prepared using the NEBNext Ultra Library Prep Kit for Illumina (New England BioLabs, Ipswich, MA, USA) and sequenced with an Illumina Hi-Seq 2500 (Illumina, San Diego, CA, USA) at the Delaware Genomics and Biotechnology Institute (Newark, DE, USA). Single-read sequencing was performed, with 150-cycle sequencing for the 3–6 and 12–15 cm samples and 50-cycle sequencing for the 24–27 cm sample. The number of reads obtained for each sample is as follows: 3–6 cm, 187,525,102; 12–15 cm, 186,874,257; and 24–27 cm, 147,990,142. Sequences are deposited in GenBank under the study PRJEB11699 (Rambo, 2016; Rambo et al., 2017).

### 16S rRNA Gene Amplicon Sequencing and Analysis

Universal 16S rRNA gene amplicons were prepared from HpaII-digested sediment core section gDNA (3–6, 12–15, and 24–27 cm) using primers 515F (GTGCCAGCMGCCGCGGTAA)/806R (GGACTACHVGGGTWTCTAAT) (Caporaso et al., 2012) and sequenced on an IonTorrent PGM (Thermo Fisher Scientific, Waltham, MA, USA). PCR preparation and sequencing was performed by Molecular Research, LP (Clearwater, Texas, USA).

Analysis of 16S rRNA gene sequences was performed with QIIME 1.8.0 (Caporaso et al., 2010). Dereplication, abundance sorting, and discarding reads less than 200 bp was performed with the USEARCH7 algorithm (Edgar, 2013). Chimeras were filtered with UCHIME (Edgar et al., 2011) using the RDP Gold Classifier training database v9 (Cole et al., 2014). Operational taxonomic unit (OTU) picking was performed at 97% similarity with UCLUST (Edgar, 2010). Non-chimeric sequences were chosen as the representative set of sequences for taxonomic assignment and alignment. Taxonomic assignments were performed with UCLUST (Edgar, 2010) using the Greengenes V13.8 database for 97% OTUs (DeSantis et al., 2006).

### Metagenome Assembly and Annotation

Metagenome sequence reads were trimmed to 51 bp and quality controlled to only include those with Phred+33 nucleotide confidence scores ≥95% using a custom Python script. Quality-controlled reads were co-assembled in IDBA (Peng et al., 2010) with parameters–mink 18–maxk 36–step 2–similar 0.97–min_count 2 (Supplementary Table S1). Phylogenetic classification of IDBA-assembled contigs was performed with PhymmBL (Brady and Salzberg, 2011) and Kraken (Wood and Salzberg, 2014). A PhymmBL identity confidence score threshold of 65% was imposed to designate higher confidence order-level assignments. Comparative taxonomic classifications were performed with Kraken (Wood and Salzberg, 2014). Contigs assigned to viral or fungal genomes in Kraken were removed from downstream analyses. Marker gene annotation of filtered contigs was performed with PhyloSift (Darling et al., 2014).

Open reading frame (ORF) prediction was performed with MetaGene (Noguchi et al., 2006). ORFs were annotated for KEGG Orthology (KO) families (Kanehisa et al., 2016) in HMMER 3.0 (Eddy, 2011) using the Functional Ontology Assignments for Metagenomes (FOAM) database (Prestat et al., 2014) and an acceptance threshold “*e*” of 1e−4. In the case of multiple KO assignments per contig, the result with the best “*e*” and bitscore was chosen to represent that contig. Contigs that did not receive a protein annotation from these software were aligned with BLASTX (Altschul et al., 1997) against the NCBI non-redundant protein database and scored with the BLOSUM62 substitution matrix (Henikoff and Henikoff, 1992), with a maximum expectation value of 1e−4 and a word size of 3.

### Metagenome CpG Methylation Quantification and Statistical Analysis

CpG methylation was calculated using a commercial bioinformatic pipeline to reconstruct probabilities of methylation at CpG sites (as used in Marsh and Pasqualone, 2014; Marsh et al., 2016; Crowgey et al., 2018; Genome Profiling LLC, Newark, DE). An overview of the pipeline workflow is available in Supplementary Figure S1. Samples were co-assembled within IDBA with the same parameters described above (Supplementary Figure S2). The methylation score metrics recovered from assembled IDBA contigs are based on independent characteristics of DNA fragmentation *via* HpaII restriction digest and random shearing. The average read coverage for all CpG scores across all samples was 13.7x. An example of a CpG coverage map is shown in Supplementary Figure S2. Computational reconstruction of CpG methylation is based on a null selection model, where the distribution of m5C modifications at any single CpG site is expected to be 50% in a large population of cells (genome copies) for CpG sites that are non-functional or silent. Where CpG methylation status is important for cellular fitness and thus there is a selection force pushing the m5C distributions away from a 50:50 equilibrium, methylation of those CpG sites among all the cellular genome copies sampled can be measured (Marsh and Pasqualone, 2014; Marsh et al., 2016; Crowgey et al., 2018).

Statistical analyses of community methylation scores were performed using R statistical package. Modalities were tested with Hartigans’ dip test for unimodality. Methylation score bootstrap standard errors (SE) and coefficients of variation (CV) were estimated (*n* = 10,000). Score variances were tested with a Brown-Forsythe Levene-type test, and normality was tested with the Shapiro-Wilk test. Two-tailed Jonckheere-Terpstra trend tests were performed with 10,000-permutation reference distributions for downcore depth trends. Kruskal-Wallis H tests were used to determine if CpG site methylation distributions were identical across depths for chitinase and transposase genes. Jonckheere-Terpstra trend tests with 10,000-permutation reference distributions (random number generator seed = 7) were performed for these same CpG site scores to determine the significance of increasing or decreasing methylation score trends with depth.

## Results

### Sediment Properties

Radionuclide dating constraints show that the Oyster Rocks site is composed of a top layer of recently deposited tidally mixed or bioturbated sediment (~4 cm, sediment age < 106 days) situated above older sediment established 50–100+ years ago (Supplementary Figure S3). Sulfate concentrations were more varied between 0–3 and 3–6 cm (Supplementary Figure S3A) for Core L, but concentrations were higher in deeper samples from 6–9 to 27–30 cm. Methane concentrations of Core L were shown to increase with depth, with higher variance between 0 and 12 cm and lower variance from 15 to 30 cm (Supplementary Figure S3B). Porosity for Core R was shown to be far lower within older sediments (Supplementary Figure S3C). While cores from separate years were used to generate sequence and geochemical data, the ages of all sediments are consistent, in that the shallowest sequenced sample is less than a year old and the deeper sequenced samples are significantly older (50+ years).

### Microbial Taxonomic Composition and Function in the Sediment

**Figure 1:**
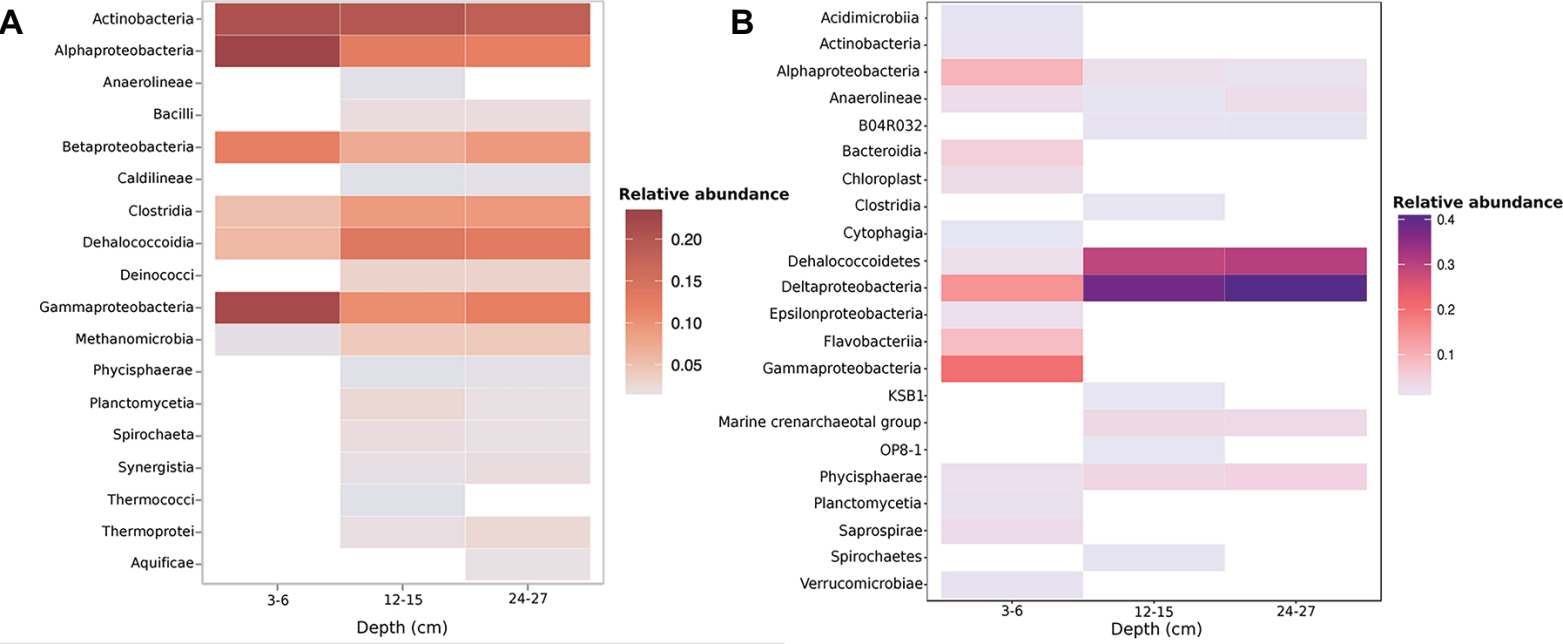
Taxonomic relative abundances of **(A)** metagenome contigs with both Kraken and higher-confidence PhymmBL class assignments present within two or more samples **(B)**. OTU class relative abundance of 16S rRNA gene amplicons. Both methods show a drastic shift in community diversity between the shallow (3–6 cm) sample and deeper (12–15 and 24–27 cm) samples.

The most abundant taxonomic classes present in metagenomic data across all depths were the *Actinobacteria*, *Bacilli*, *Clostridia*, *Dehalococcoidia*, and α-β-δ-γ-*Proteobacteria* (Figure 1, Supplementary Figure S5). Metagenomic data indicated a prevalence of anaerobic taxa within the 12–15 and 24–27 cm samples, with the presence of methanogenic archaea *Methanomicrobia* and *Dehalococcoidia* within the 12–15 and 24–27 cm samples (Figure 1A). KO annotations suggest the functional potential for anaerobic metabolism at depth (Supplementary Figure S6). The deeper samples also had a higher presence of *Dehalococcoidetes* and *Deltaproteobacteria*, as well as Marine Crenarchaeota Group and Marine Hydrothermal Vent Group archaea as evidenced by amplicon sequencing of the 16S rRNA gene (Figure 1B). OTUs were clearly shared between the three depths, and the diversity of 16S rRNA genes was generally higher at 3–6 cm than the 12–15 and 24–27 cm samples (Supplementary Figure S4).

### Metagenome CpG Methylation

From these metagenome data, we assessed the methylation states of CpG sites (example in Supplementary Figure S2). A total of 6,254 CpG sites that could be directly compared between all three samples were mapped to 3,743 contigs (4.33% of all three unprocessed IDBA assemblies). Differential methylation states between depths were observed in 1,173 CpG sites, while the remaining 5,081 had equivalent methylation states. A total of 4,235 (67.7%) of these CpG sites were identified within contigs receiving higher-confidence PhymmBL order-level classifications.

**Figure 2:**
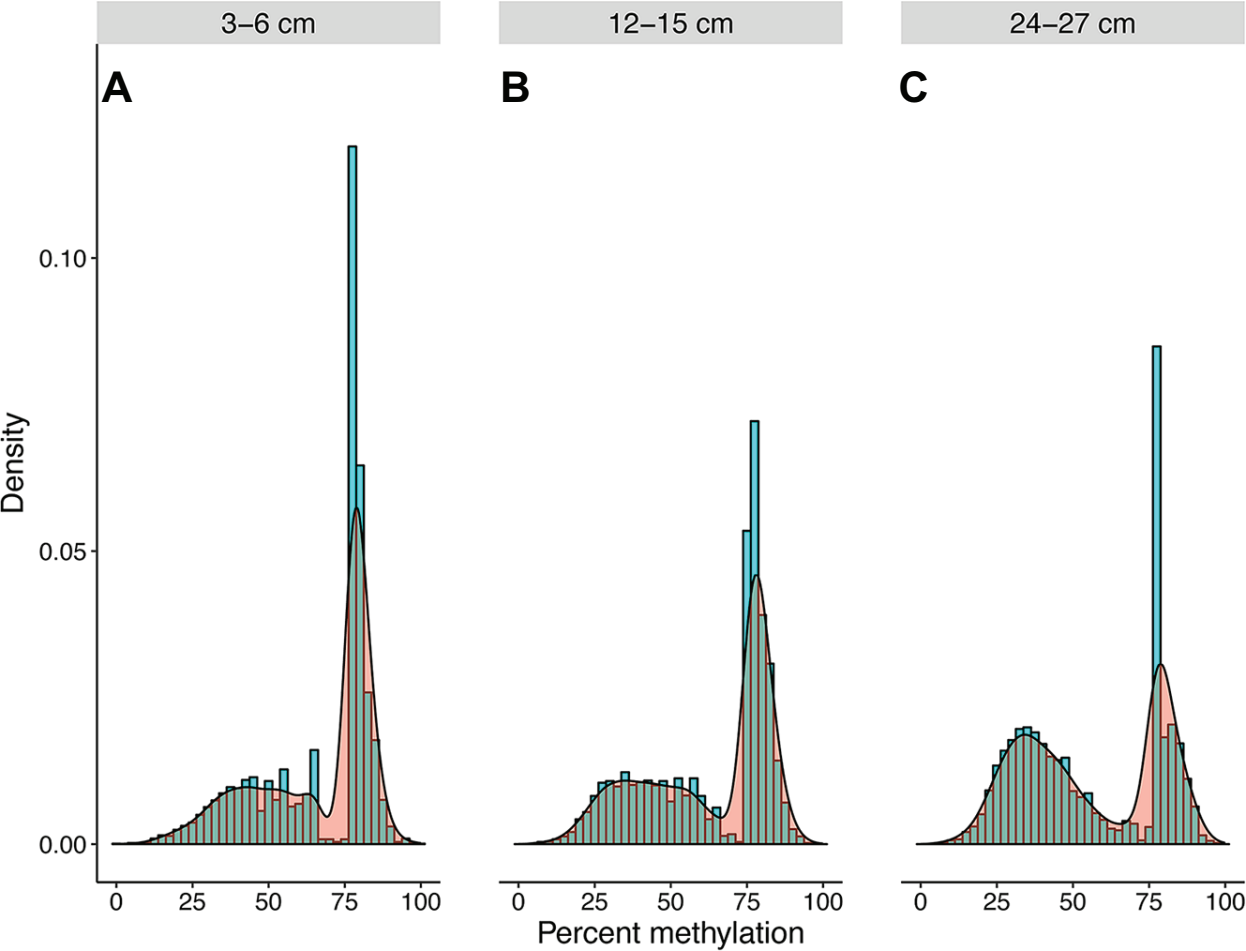
Community methylation load distributions. Histograms and kernel density overlays represent overall methylation levels of CpG sites that are shared across all three samples. A greater number of recovered CpG sites were highly methylated at 3–6 cm **(A)**, resulting in a greater methylation load at this depth. However, a trend of decreasing overall methylation was seen at 12–15 cm **(B)** and 24–27 cm **(C)**, with more sites experiencing transitions from highly methylated to non-methylated or lower-methylated states or persisting in non-methylated states.

**Figure 3:**
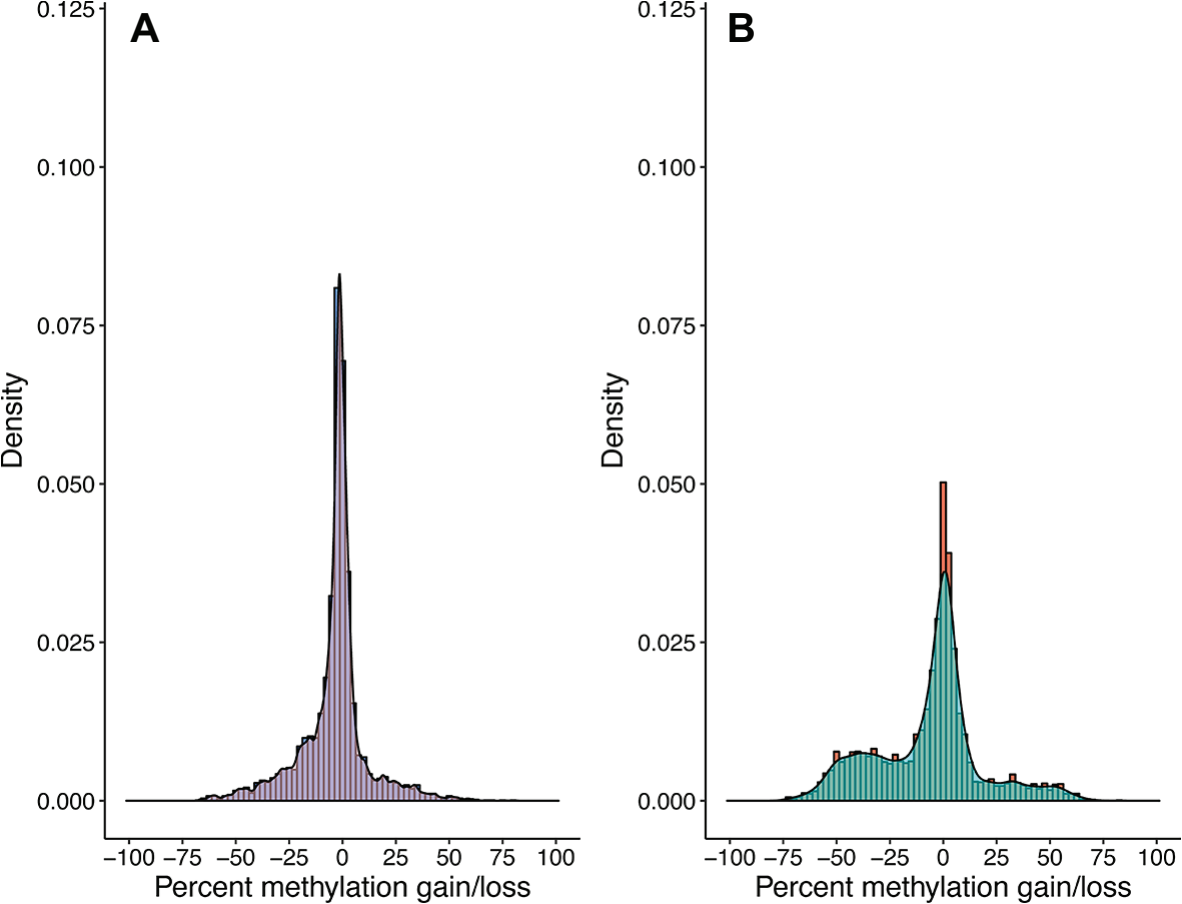
CpG methylation gains and losses from **(A)** 3–6 to 12–15 cm and **(B)** 12–15 to 24–27 cm. Histograms and kernel density overlays are representative of the densities of methylation shifts for individual CpG sites. Sites represented in **(A)** are the same sites represented in **(B)**. Shifts range from −100 (total methylation loss) to +100 (total methylation gain). A significant number of CpG sites remained at equivalent methylation states from 3–6 to 12–15 cm, yet there is a decrease in methylation ranging from ~25 to ~50% from 12–15 to 24–27 cm.

**Figure 4:**
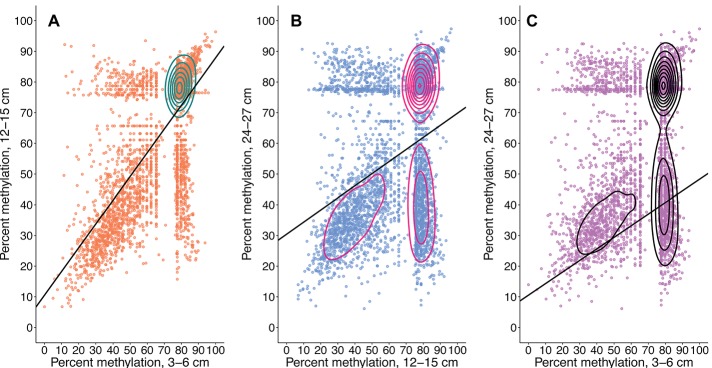
Total CpG methylation shifts from 3–6 to 12–15 cm **(A)**, 12–15 to 24–27 cm **(B)**, and 3–6 to 24–27 cm **(C)**. Plots are representative of metagenome-wide methylation loads. Each point is a recovered CpG site whose quantified methylation states are comparable across all three samples. The CpG sites represented in **(A)** are the same as those represented in **(B)** and **(C)**. Contours were calculated by 2D kernel density estimation. Changes in a CpG site’s methylation profile can be traced from a shallower sample (x-axis) to a deeper sample (y-axis). A greater number of sites remain in highly methylated states from 3–6 to 12–15 cm. These same CpG sites experience a general trend of methylation loss from the mid sample to the deepest sample; a similar trend is observed from 3–6 to 24–27 cm. An increased number of CpG sites with methylation loads ~80% at 12–15 cm undergo methylation losses ranging from 20–60% upon transitioning to 24–27 cm. Linear equations, *R*^2^, and *p*’s are as follows: **(A)** *y* = 0.77331x + 10.54275, *R*^2^ = 0.48707, *p* = 1.635522e-52; **(B)** *y* = 0.39383x + 30.42409, *R*^2^ = 0.14011, *p* = 5.221187e-285; and **(C)** *y* = 0.37748x + 29.80657, *R*^2^ = 0.10484, *p* = 4.739596e-200.

Community-wide methylation distributions showed that many CpG sites remain in highly methylated states from 3–6 to 12–15 cm (Figures 2A, 3A). An overall decrease in methylation percentage was seen at 12–15 cm (Figure 2B) and 24–27 cm (Figure 2C), with CpG sites exhibiting more equilibrated, bimodal states of high and low methylation. A decrease in methylation, ranging from ~25 to 50% was shown to account for the bimodal state (Figure 3B), with higher numbers of sites shifting from ~80 to 90% methylated states to lower-methylated and non-methylated states (Figure 4B). A greater number of CpG sites experienced shifts from non-methylated states to fully methylated states when transitioning from 12–15 to 24–27 cm, potentially indicating *de novo* methylation of these sites. However, many CpG sites were shown to remain in non-methylated states between 12–15 and 24–27 cm.

The methylation dynamics of individual CpG sites were analyzed for the taxa that were most abundant within the CpG profiles. An overall trend of increasing methylation score standard error (SE) and coefficient of variation (CV) with depth was seen in all analyzed phyla (Supplementary Table S2). There is a general trend of decreasing CV for methylation scores with depth influenced by an overall trend toward bimodal score distributions. Hartigans’ dip test results support a non-unimodal distribution of methylation scores for analyzed phyla (Supplementary Table S3), verifying mixed methylation profiles. Brown-Forsythe tests suggest that CpG score variances across depths were unequal for 70% of analyzed phyla (*p* < 0.05), supporting the presence of mixed methylation profiles and dynamic shifts in methylation states (Supplementary Table S4). Methylation scores for the majority of phyla exhibit decreasing trends with depth (Supplementary Table S4; Figures 2, 4).

**Figure 5:**
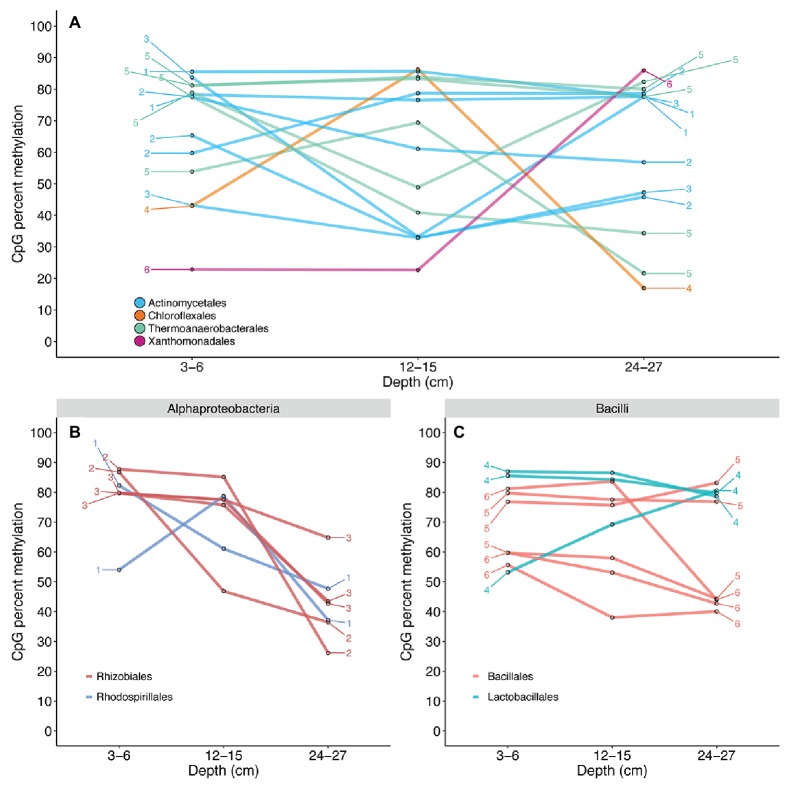
CpG methylation dynamics of chitinase **(A)** and transposase **(B,C)** genes. Connected points are representative of changes in the methylation state of a single CpG site across the depths of each sample. Numbers denote contigs to which these CpG sites are mapped; multiple CpG sites exhibiting differential methylation within the same gene were recovered for the Actinomycetales and Thermoanaerobacterales. The Actinomycetales appear to maintain relatively similar chitinase CpG methylation. The Thermoanaerobacterales exhibit more drastic methylation losses with depth; however, a significant trend of methylation loss was not established (Supplementary Table S5). The recovered CpG site mapped to Xanthomonadales was shown to persist in a non-methylated state from 3–6 to 12–15 cm, but exhibited a possible *de novo* methylation event from 12–15 to 24–27 cm. CpG sites for transposase genes mapped to Alphaproteobacteria **(B)** and Bacilli **(C)** were shown to exist in differential methylation states on the same contig across depths. The Alphaproteobacteria exhibit a general decrease in CpG methylation with depth, while Bacilli suggests slightly decreasing, but relatively stable methylation across depths. The methylation states of multiple CpG sites located within the same contig were shown to shift to highly similar states in several instances.

Contigs receiving reliable KEGG Orthology annotations contained only 35 CpG sites. Of these 35 CpG sites, all intergenic, chitinase gene annotations were recovered for 14 comparable sites that could be traced back to six contigs with higher-confidence PhymmBL classifications (Figure 5A **)**. A total of 12 quantifiable sites exhibiting differential methylation states within the same gene were identified for *Actinomycetales* and *Thermoanaerobacterales*. Quantifiable states of 97 CpG sites were recovered for transposase genes identified by BLASTX alignments. Transposase CpG sites that were methylated in surface samples tend to remain in methylated states across depths, although several methylated sites undergo shifts into lower-methylated or non-methylated sites in deeper samples for the *Alphaproteobacteria* (Figure 5B). Transposase CpG sites for the *Bacilli* appear to generally remain at highly methylated or lower-methylated states (Figure 5C). CpG sites existing within the same contig tend to shift to similar methylation states from 12–15 to 24–27 cm. Kruskal-Wallis H test and Jonckheere-Terpstra trend test results suggest that CpG methylation score distributions are identical across depths for recovered chitinase (*Actinobacteria*, *Clostridia*) and transposase (*Alphaproteobacteria*, *Actinobacteria*, *Gammaproteobacteria*, *Bacilli*, and *Betaproteobacteria*) genes and do not exhibit significant increasing or decreasing methylation score trends with depth (Supplementary Table S5).

## Discussion

Shifts in community composition observed through both 16S rRNA gene and metagenomic gDNA sequencing appear to be related to the drastic change in sediment age suggested by radionuclide constraints (Supplementary Figures S3–S6). The 3–6 cm sample encompasses the transition zone from young, fresh sediment to older, established sediment at 4–5 and 5–6 cm, so overlap in communities was expected. Our results clearly show increases in age from 3–6 to 12–15 cm, and the deeper depth of 24–27 cm is certainly older although our tests could not measure an exact age between 15 and 24 cm. The results support the presence of a shifting downcore age gradient with taxa associated with facultative and obligate anaerobic metabolic strategies being present at greater abundance with depth (Figure 1). A discrepancy in the relative abundance of *Actinobacteria* and *Deltaproteobacteria* sequences exists between 16S rRNA gene amplicon and metagenome results. *Actinobacteria* are shown to have high relative abundance through all three depths in the metagenome (Figure 1A), yet amplicons show *Actinobacteria* present only in the 3–6 cm sample in lower abundances (Figure 1B). This could be due to HpaII-digested DNA being used in 16S rRNA gene amplicon libraries, as Actinomycetes have been shown to possess multiple HpaII target sites within their 16S rRNA genes (Tolba et al., 2013). *Deltaproteobacteria* were seen in the 16S rRNA gene amplicons, but not in the metagenome by Kraken annotation, but were observed by PhyloSift marker gene examination (Figure 2, Supplementary Figure S5). Yet, both methods show an increase in anaerobic taxa and shared organisms across all three depths. CpG methylation profiles recovered from these sediments were mapped to taxa and genes, with individual CpG sites exhibiting shifts toward higher or lower proportional methylation states across this sediment boundary.

Of the CpG site populations assigned to taxa, 22% had <5% methylation gains or losses between depths. While these sites remained within generally equivalent states, 9.22% of recovered CpGs were observed to have differential methylation shifts ≥50% between depths (Figures 2, 3). This may be representative of the standard binary response associated with the concept of an epigenetic on/off switch (Hernday et al., 2004; Marsh and Pasqualone, 2014). Shifts between highly methylated and fractionally methylated states suggest the presence of dynamic CpG sites that contribute to a mixed population. However, we cannot rule out the potential effect of gene or whole genome duplication on methylation scoring, as newly replicated DNA would contain fewer methylated bases and is highly dependent upon maintenance methylation (Casadesús and Low, 2006). We additionally cannot detect variance within a population, so quantifying strain-level CpG methylation among growing or senescent populations would be difficult to measure with this method. However, theory states that cellular growth, and therefore creation of new DNA, should slow in older sediments with the decrease in cell turnover (Hoehler and Jørgensen, 2013). *Escherichia coli* research has shown that stationary phase, older cells should contain a higher abundance of cytosine methylated bases (Kahramanoglou et al., 2012) and nutrient-starved cells will maintain their adenine methylation (Westphal et al., 2016); hence the finding of decreasing methylation downcore goes contrary to theoretical expectations (Figure 3). Abiotic decay of extracellular DNA (eDNA) could account for some of this signal, since the respective hydrolytic deamination of cytosine and 5mC to uracil and thiamine in eDNA may account for decreased CpG methylation at depth. However, since we see specific genes increase and decrease their methylation scores, eDNA is not likely to be responsible for the majority of our detected signal.

CpG methylation states were shown to vary for specific genes including chitinases (Figure 5A) and transposases (Figures 5B,C). Chitinolytic bacteria are widely distributed in sediment environments (Bhattacharya et al., 2007; Souza et al., 2011) and are responsible for converting this insoluble source of carbon and nitrogen into a widely used form. It has been previously noted that chitin is rapidly removed from an estuary within the first 10 cm of sediment (Gooday, 1990). The most abundant shifts from the 3–6 to 12–15 cm samples are from methylated to unmethylated states within lineages of *Actinobacteria* and *Thermoanaerobacter* (Figure 5A). However, significant, unidirectional decreasing or increasing CpG methylation trends across depths were not supported (Supplementary Table S5). Multiple CpG sites on the same contig were shown to remain at methylated or non-methylated states across depths (Figure 5A, contig 1) or exhibit similar scores at the shallow (3–6 cm) and deep (24–27 cm) depths, yet tend to undergo methylation gains or losses at mid-depth (12–15 cm; e.g., Figure 5A, contig 5). We hypothesize the variations in these signals to suggest regulation, and not just a signature of cellular replication, considering that methylation losses are greater upon transition to the anaerobic 12–15 cm depth, but this would need to be proven with transcript or enzyme-based experiments. However, these same CpG sites are again methylated within the 24–27 cm depth, as they leave the assumed zone of available chitin. While chitin was not concurrently measured, the often noted correlation between cultivable chitinolytic bacteria and chitin abundances suggests that this process is one that would not be maintained if chitin were not present (Gooday, 1990). The evidence for anaerobic organisms only reducing methylation from chitinase CpG sites within the anaerobic sedimentary horizons suggests that this is methylation-based regulation of an metabolically energetically costly process. We postulate that this is an initial glimpse into how marine sediment microbes potentially utilize DNA methylation to regulate biogeochemical processes that are vital for nutrient cycling.

We also show differential methylation of multiple CpG sites within sediment bacterial transposase genes (Figures 5B,C). Similar to recovered chitinase CpGs, significant decreasing or increasing methylation trends were not observed (Supplementary Table S5). CpG sites on the same contig were shown to remain methylated from 3–6 to 12–15 cm, while undergoing demethylation (Figure 5B, contig 2) or re-methylation (Figure 5C, contig 4) at 24–27 cm. Transposase regulation has been observed to take place *via* adenine methylation at promoter GATC sites in *Escherichia coli* (Roberts et al., 1985; Yin et al., 1988; Dodson and Berg, 1989), yet the regulatory mechanisms of one model organism do not necessarily apply to the entire bacterial domain. To our knowledge, this is the first suggestion of cytosine methylation-based regulation of transposases in bacteria; however, our data do not distinguish between activation and quiescence of the signal. The regulation of transposases and transposon mobility could play a role in rapid acclimation responses by influencing transcriptional activity and the ability for mobile elements to be inserted into a genome. Due to the known influence of DNA methylation within bacterial transposases and the results of this study, we speculate that CpG methylation could act as a regulator of transposition within sediment.

This preliminary study includes caveats, in that there is a lack of replication and the need for demonstration of our suggested regulatory mechanisms in a laboratory setting. Another consideration is that shotgun metagenomic sequencing of environmental samples accounts for bulk DNA within a community where individual cells likely vary in their physiological states and genomic methylation profiles, something that may be able to be distinguished with further sequencing advances. However, the proof-of-principle study presented here shows that it is possible to detect complex methylation signatures within a mixed metagenome and that, as organisms experience anoxia and increased age within sediment, their methylation profiles change. The literature-based expectation would be an increase in methylation with the aging of a cell, yet we detected many shifts to a non-methylated state (Figure 2). As such, we hypothesize this is a regulatory signature that may be employed during low-energy growth typical in aged sediments (Hoehler and Jørgensen, 2013). Yet, since this is a tidally influenced system where fluid mixing may occur and energy stress is not nearly as heavy as a truly deep biosphere, this remains as conjecture.

As epigenetic research shifts from model systems toward potentially novel organisms within natural environments, there is a need for the development of assays capable of detecting epigenetic signatures within environmental samples (Walworth et al., 2017). This study provides a community-level insight into the dynamic behavior of CpG methylation sites within estuarine sediments. A benefit of this Illumina assay is that it requires less DNA than single-molecule approaches and allows for CpG site mapping to specific taxa and genes. Ongoing modifications tailored for metagenomic samples will pave the way for the reconstruction of dynamic methylation profiles within genomes obtained from the environment and a better understanding of potential regulatory influences of microbial epigenetics.

## Author Contributions

IR performed the experiments, analyzed the data, and wrote the manuscript. AM and JB conceived of the project, analyzed the data, and wrote the manuscript.

### Conflict of Interest Statement

The software platform designed for processing DNA methylation profiles from NGS sequence data is licensed from the University of Delaware to Genome Profiling LLC, a company co-founded by AM and developed with support from an Innovation Corps Grant from the National Science Foundation to AM (NSF1355306). IR and JB declare no financial involvement with any commercial entity.
