# Supplementary material for: Cytosine Methylation Within Marine Sediment Microbial Communities: Potential Epigenetic Adaptation to the Environment
Source: Front Microbiol. 2019 Jun 11;10:1291. doi: 10.3389/fmicb.2019.01291 (PMC6579885; doi:10.3389/fmicb.2019.01291)
Supplement: Supplementary file 1 [file Data_Sheet_1.docx]

**Supplemental Figures**


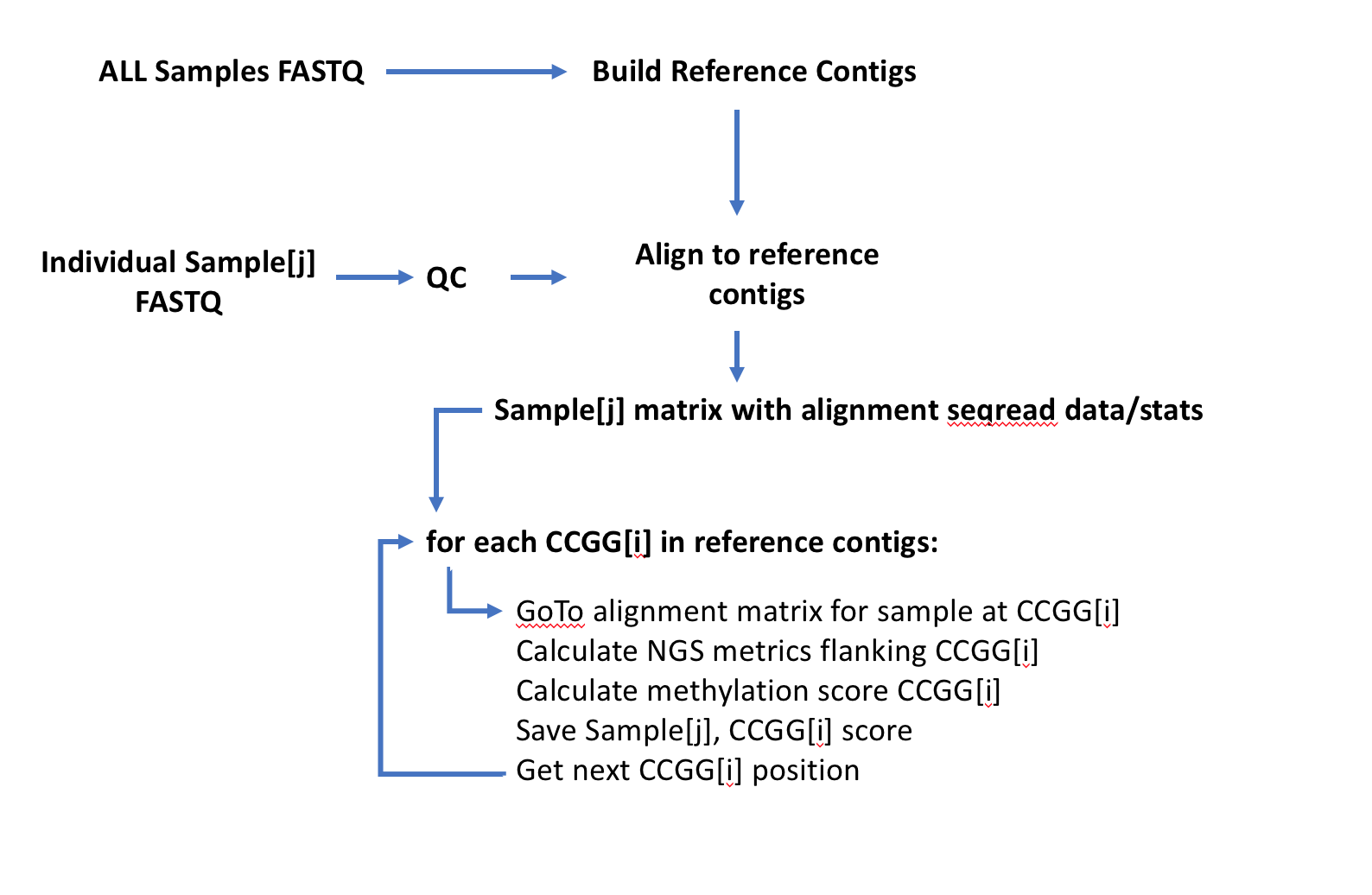


**Figure S1**: Outline of CpG methylation scoring algorithm workflow. The calculation of a methylation score value is executed for each individual CpG site (in a CCGG context) that has been identified in the reference contigs for a project. The calculation uses a local domain approach to estimate over- or under-representation bias of the CCGG[i] site in the compiled sequence reads. Several metrics are derived for that local region and the methylation status of the CpG site is assessed via a ratio, or beta score, where a value of 1.0 is the result when all gDNA copies of that CpG site are methylated and 0.0 is the result when they are all unmethylated. Fractional scores between 0.0 and 1.0 indicate heterogenous methylation and can be interpreted as the percentage of gDNA fragments methylated at that site relative to the total number of gDNA fragments covering that site in the sample.

**
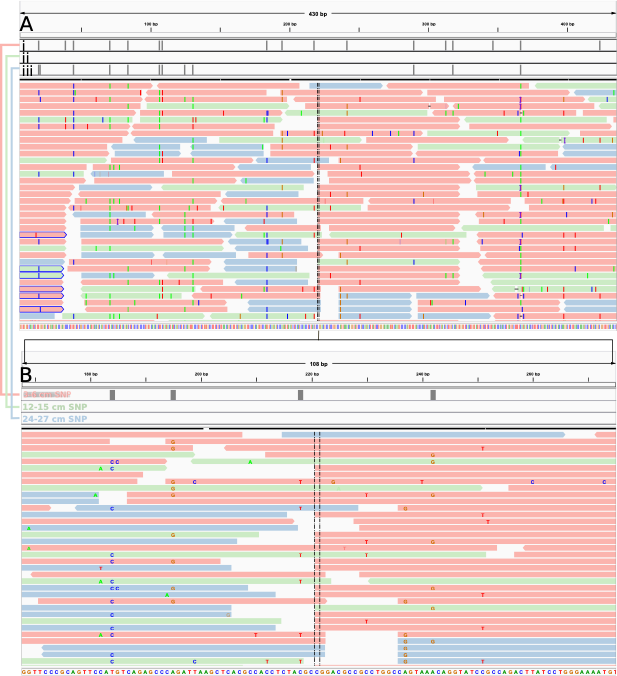
**

**Figure S2**: Mapping of methylated and non-methylated reads to combined assembly CCGG site. Panels show **(A)** expanded and **(B)** zoomed read overlaps for a contig portion containing a single CCGG site of sufficient coverage for methylation scoring. Reads were mapped to the combined IDBA assembly using bwa mem (Li 2013). SNP calling was performed with bcftools and vcfutils.pl within the samtools software suite (Li et al., 2009). Tracks i, ii, and iii represent SNP calls for 3-6 cm, 12-15 cm, and 24-27 cm reads, respectively. SNPs are identified by vertical gray bars. Reads are grouped by color (red = 3-6 cm, green = 12-15 cm, blue = 24-27 cm). Reads with right-facing arrows indicate (+) strands, while those with left-facing arrows indicate (-) strands. Reads starting at HpaII cut sites (5’-C^CGG-3’; 3’-GGC^C-5’) indicate non-methylated cytosines, while intact CpG sites indicate methylated cytosines. Reference contigs are assembled from the pooled sequence reads from all samples. The probability of a CpG site being methylated is calculated by re-aligning the sequence reads from each sample independently to these reference contigs.


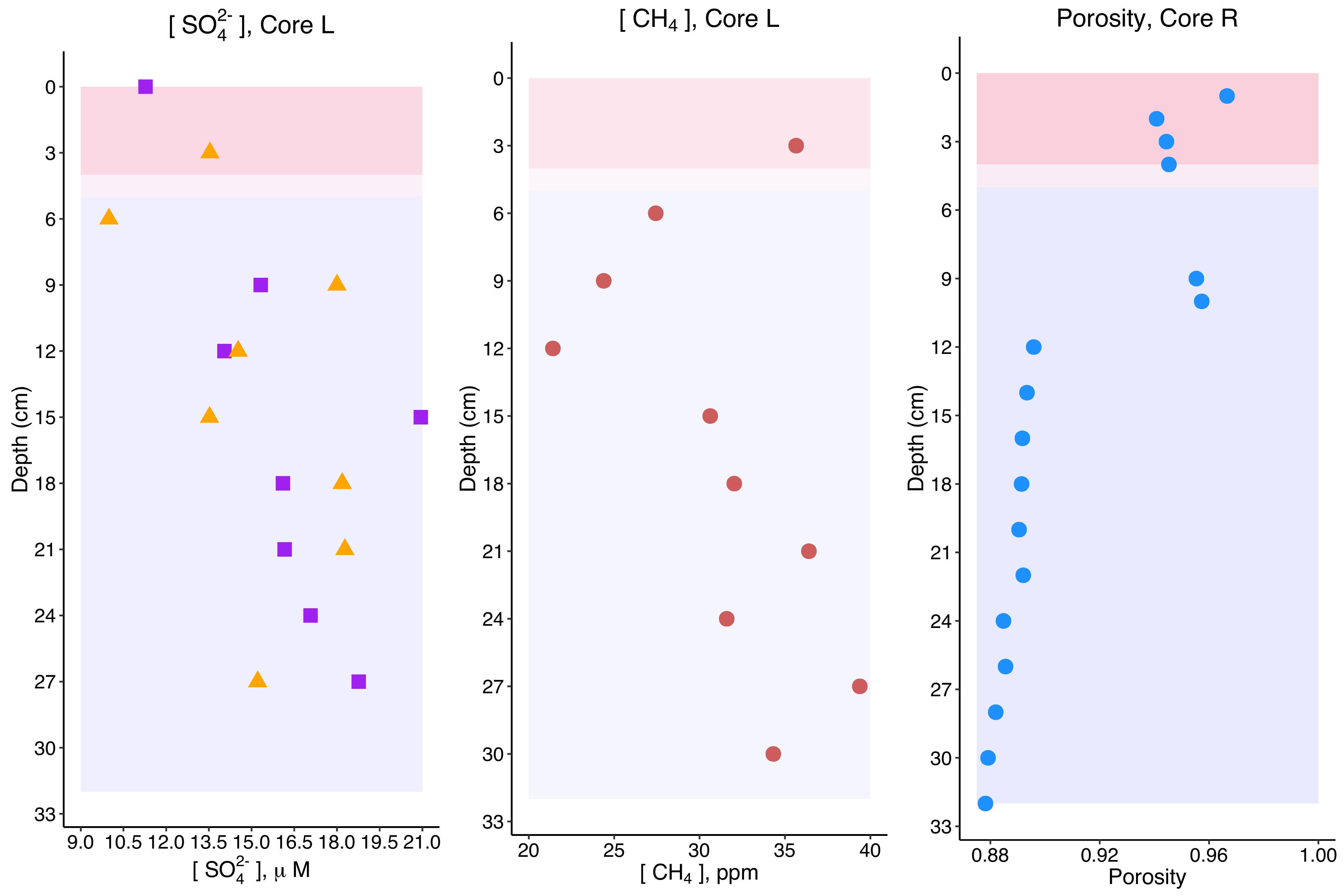


**Figure S3**: Sediment core sulfate **(A)**, methane **(B)**, and porosity **(C)**. Sulfate (purple squares = 1:6 dilution, orange triangles = 1:11 dilution) and methane concentrations are displayed for Core L. Porosity is displayed for Core R. Shaded bars are representative of radionuclide age constraints along the depth of Core R (peach = youngest sediment, age < 106 days; pink = extent of ^210^Pb from 0 cm to transition zone; blue = sediment 50-100+ years old).

**Figure S4**: Rarefied Chao1 diversity index for 16S rRNA gene OTU tables. Community diversity is higher within recently deposited surface sediments (3-6 cm) and lower within older, established sediments (12-15 cm, 24-27 cm).


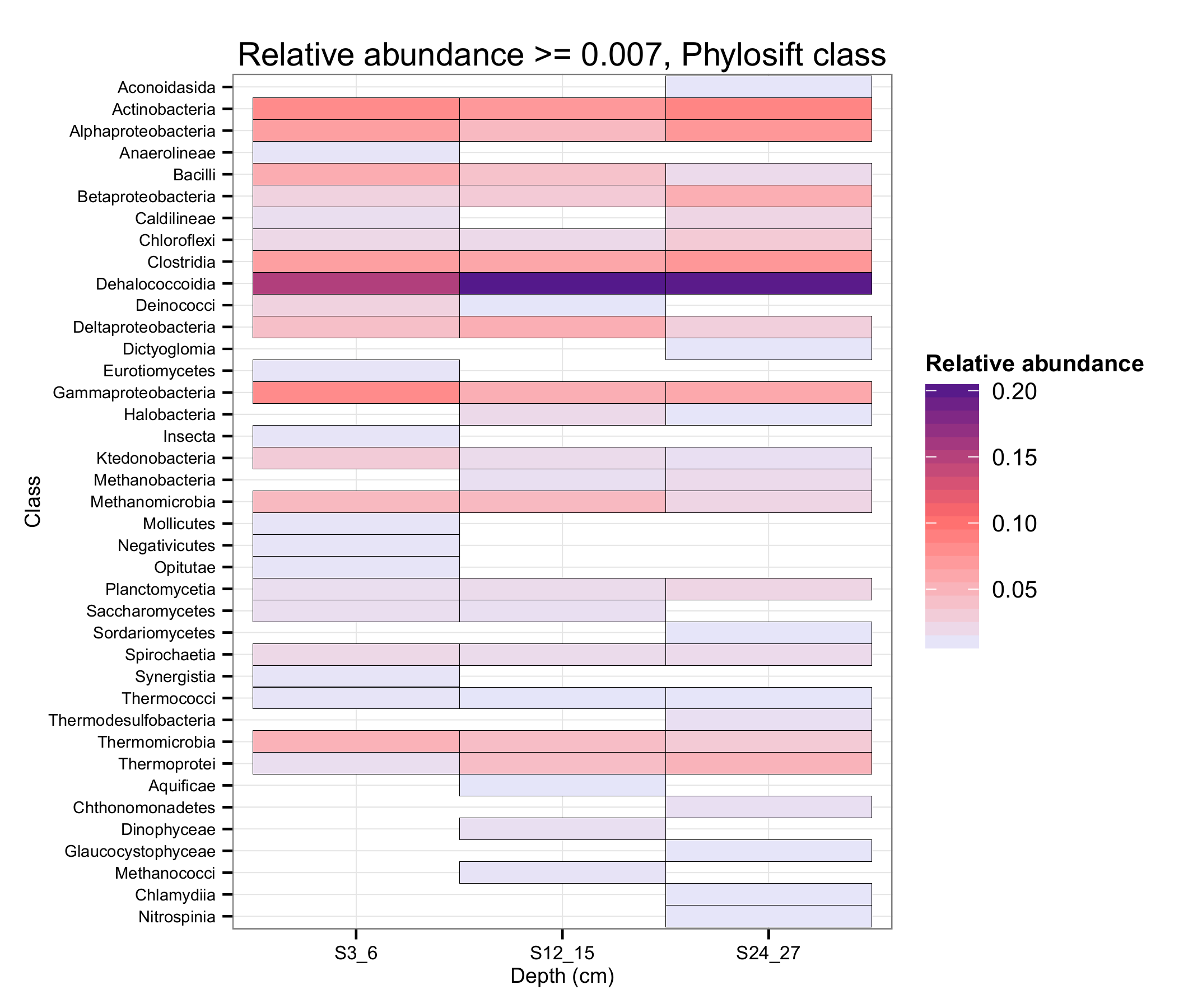


**Figure S5**: Class-level abundance of marker genes annotated with Phylosift. Marker gene annotation suggests an increased abundance of several anaerobic classes in deeper samples, which could be indicative of a more anaerobic sediment environment at 12-15 cm and 24-27 cm.

**Figure S6**: Genes for anaerobic metabolism (methane and sulfur-related) seen across depths, according to KO annotation. Genes are separated into those involved in sulfur metabolism (KEGG map00920) and methane metabolism (KEGG map00680). Relative abundance values were standardized in R via Hellinger transformation with the vegan package (Oksanen et al., 2018). Greater abundances of genes involved in methanogenesis (trimethylamine corrinoid protein co-methyltransferase) were present within the 24-27 cm sample. Genes involved in assimilatory sulfate reduction were identified (sulfate adenylyltransferase (*sat*), adenylylsulfate kinase (*cysC*), phosphoadenosine phosphosulfate reductase (*cysH*)) as well as the sulfite reductase beta subunit (*dsrB*) involved in dissimilatory sulfate reduction, however apparent downcore trends of increased sulfate reduction potential were not observed.

Oksanen, J., Blanchet, F.G., Friendly, M., Kindt, R., Legendre, P., McGlinn, D.,

et al. (2018). vegan: Community ecology package. R package version 2.4-6.

Available at: https://CRAN.R-project.org/package=vegan

**Supplemental Tables**

| **Table S1**: IDBA assembly statistics | |  |  |  |  |
| --- | --- | --- | --- | --- | --- |
|  | Total | High Abundance | Low Abundance | Singletons | Viral contigs |
| 3-6 cm |  |  |  |  |  |
| Number of contigs | 18800 | 4617 | 191 | 13647 | 345 |
| Number of bases | 2,493,769 | 1,214,155 | 283,733 | 917,933 | 77,948 |
| Minimum length | 34 | 200 | 200 | 34 | 34 |
| Maximum length | 5808 | 3919 | 342 | 199 | 5808 |
| Median length | 73 | 279 | 248 | 54 | 162 |
| Mean length | 132 | 314 | 252 | 67 | 226 |
| N50 contig length | 251 | 299 | 249 | 75 | 336 |
| N80 contig length | 79 | 251 | 238 | 45 | 250 |
| 12-15 cm |  |  |  |  |  |
| Number of contigs | 49214 | 13815 | 3386 | 31538 | 475 |
| Number of bases | 8,218,771 | 4,841,641 | 900,041 | 2,319,312 | 157,777 |
| Minimum length | 34 | 200 | 200 | 34 | 35 |
| Maximum length | 5419 | 2782 | 484 | 281 | 5419 |
| Median length | 95 | 309 | 258 | 65 | 296 |
| Mean length | 167 | 350 | 266 | 73 | 332 |
| N50 contig length | 276 | 346 | 261 | 83 | 334 |
| N80 contig length | 112 | 267 | 241 | 50 | 264 |
| 24-27 cm |  |  |  |  |  |
| Number of contigs | 18361 | 4010 | 1114 | 13105 | 132 |
| Number of bases | 2,474,539 | 1,238,974 | 295,037 | 893,040 | 47,488 |
| Minimum length | 34 | 200 | 201 | 34 | 35 |
| Maximum length | 5419 | 995 | 429 | 259 | 5419 |
| Median length | 76 | 282 | 256 | 53 | 287 |
| Mean length | 134 | 309 | 265 | 68 | 360 |
| N50 contig length | 250 | 301 | 260 | 76 | 328 |
| N80 contig length | 83 | 252 | 241 | 46 | 269 |

| **Table S2**: Bootstrapped (n=10,000) methylation score standard errors (SE) and coefficients of variation (CV) | | | |
| --- | --- | --- | --- |
| Phylum | Depth (cm) | Bootstrap SE | Bootstrap CV |
| Actinobacteria | 3-6 | 0.2657 | 0.0192 |
|  | 12-15 | 0.2285 | 0.0167 |
|  | 24-27 | 0.1629 | 0.0118 |
| Bacteroidetes | 3-6 | 0.7011 | 0.0607 |
|  | 12-15 | 0.7138 | 0.061 |
|  | 24-27 | 0.5838 | 0.0409 |
| Chloroflexi | 3-6 | 0.7011 | 0.0571 |
|  | 12-15 | 0.6841 | 0.0585 |
|  | 24-27 | 0.6902 | 0.058 |
| Crenarchaeota | 3-6 | 0.788 | 0.0625 |
|  | 12-15 | 0.9782 | 0.0756 |
|  | 24-27 | 0.7357 | 0.0554 |
| Cyanobacteria | 3-6 | 0.9101 | 0.0663 |
|  | 12-15 | 1.2642 | 0.0929 |
|  | 24-27 | 1.0201 | 0.0867 |
| Deinococcus-Thermus | 3-6 | 1.2122 | 0.0881 |
|  | 12-15 | 1.247 | 0.0944 |
|  | 24-27 | 0.9514 | 0.0804 |
| Euryarchaeota | 3-6 | 1.1555 | 0.0826 |
|  | 12-15 | 1.2405 | 0.0893 |
|  | 24-27 | 0.9118 | 0.0758 |
| Firmicutes | 3-6 | 1.1583 | 0.084 |
|  | 12-15 | 1.2099 | 0.0878 |
|  | 24-27 | 0.9922 | 0.0804 |
| Proteobacteria | 3-6 | 1.0983 | 0.0793 |
|  | 12-15 | 1.1427 | 0.0829 |
|  | 24-27 | 0.9387 | 0.0768 |
| Spirochaetes | 3-6 | 1.1197 | 0.0771 |
|  | 12-15 | 1.1119 | 0.0879 |
|  | 24-27 | 0.9764 | 0.0753 |

| **Table S3**: Hartigans’ dip test for unimodality results | | | |
| --- | --- | --- | --- |
| **Phylum** | **Depth (cm)** | **D statistic** | **p-value** |
| **Actinobacteria** | 3-6 | 0.050313 | < 2.2e-16 |
|  | 12-15 | 0.034191 | 2.93E-06 |
|  | 24-27 | 0.072555 | < 2.2e-16 |
| **Bacteroidetes** | 3-6 | 0.053424 | 0.1125 |
|  | 12-15 | 0.074252 | 0.002792 |
|  | 24-27 | 0.086669 | 0.0001283 |
| **Chloroflexi** | 3-6 | 0.049039 | 0.009716 |
|  | 12-15 | 0.04698 | 0.01705 |
|  | 24-27 | 0.078279 | 5.10E-07 |
| **Crenarchaeota** | 3-6 | 0.063378 | 0.05662 |
|  | 12-15 | 0.066014 | 0.03983 |
|  | 24-27 | 0.13102 | < 2.2e-16 |
| **Cyanobacteria** | 3-6 | 0.047953 | 0.02808 |
|  | 12-15 | 0.062959 | 0.0004542 |
|  | 24-27 | 0.11856 | < 2.2e-16 |
| **Deinococcus-Thermus** | 3-6 | 0.080579 | 5.36E-06 |
|  | 12-15 | 0.057434 | 0.00436 |
|  | 24-27 | 0.08857 | < 2.2e-16 |
| **Euryarchaeota** | 3-6 | 0.059513 | 1.13E-06 |
|  | 12-15 | 0.046572 | 0.000234 |
|  | 24-27 | 0.10763 | < 2.2e-16 |
| **Firmicutes** | 3-6 | 0.051648 | < 2.2e-16 |
|  | 12-15 | 0.041324 | 1.25E-05 |
|  | 24-27 | 0.074834 | < 2.2e-16 |
| **Proteobacteria** | 3-6 | 0.039581 | < 2.2e-16 |
|  | 12-15 | 0.039889 | < 2.2e-16 |
|  | 24-27 | 0.080087 | < 2.2e-16 |
| **Spirochaetes** | 3-6 | 0.06179 | 0.06803 |
|  | 12-15 | 0.074316 | 0.008778 |
|  | 24-27 | 0.12886 | < 2.2e-16 |

| **Table S4**: Two-tailed Jonckheere-Terpstra trend test and Brown-Forsythe variance test results for recovered CpG methylation scores | | | | |
| --- | --- | --- | --- | --- |
|  |  |  |  |  |
| Phylum | Jonckheere-Terpstra | | Brown-Forsythe | |
|  | Test statistic | p-value | Test statistic | p-value |
| Actinobacteria | 746200 | 2.00E-04 | 53.461 | < 2.2e-16 |
| Bacteroidetes | 7212 | 0.0526 | 1.4017 | 0.2484 |
| Chloroflexi | 26754 | 2.00E-04 | 8.8084 | 0.0001772 |
| Crenarchaeota | 4681 | 0.0176 | 4.944 | 0.008124 |
| Cyanobacteria | 19950 | 2.00E-04 | 3.4853 | 0.0316 |
| Deinococcus-Thermus | 17766 | 2.00E-04 | 2.2334 | 0.1086 |
| Euryarchaeota | 83664 | 2.00E-04 | 15.28 | 3.07E-07 |
| Firmicutes | 255060 | 2.00E-04 | 26.025 | 8.09E-12 |
| Proteobacteria | 4638500 | 2.00E-04 | 75.051 | < 2.2e-16 |
| Spirochaetes | 4928.5 | 0.0348 | 2.2521 | 0.1081 |

**Table S5**: Jonckheere-Terpstra trend test (T = test statistic; p_i_ = p-value, alternative hypothesis = increasing trend; p_d_ = p-value, alternative hypothesis = decreasing trend) and Kruskall-Wallis H test results for transposase and chitinase CpG site (nCpG) methylation scores.

| Gene | Class | nCpG | Jonckheere-Terpstra | | | Kruskall-Wallis |
| --- | --- | --- | --- | --- | --- | --- |
|  |  |  | T | p_i_ | p_d_ | p-value |
| Transposase | Alphaproteobacteria | 13 | 201 | 0.9106 | 0.0868 | 0.3743 |
|  | Actinobacteria | 22 | 718 | 0.5374 | 0.4704 | 0.8156 |
|  | Gammaproteobacteria | 32 | 1313 | 0.9289 | 0.0636 | 0.2826 |
|  | Bacilli | 13 | 222 | 0.7895 | 0.2114 | 0.6527 |
|  | Betaproteobacteria | 17 | 349 | 0.9261 | 0.0728 | 0.1381 |
| Chitinase | Actinobacteria | 7 | 66 | 0.6806 | 0.3222 | 0.5859 |
|  | Clostridia | 5 | 30 | 0.7831 | 0.2205 | 0.7554 |
